# Bioadhesive and Injectable Hydrogels and Their Correlation with Mesenchymal Stem Cells Differentiation for Cartilage Repair: A Mini-Review

**DOI:** 10.3390/polym15214228

**Published:** 2023-10-26

**Authors:** Ján Kováč, Petra Priščáková, Helena Gbelcová, Abolfazl Heydari, Stanislav Žiaran

**Affiliations:** 1 Medical Vision, Záhradnícka 55, 821 08 Bratislava, Slovakia; jan.kovac@nurch.sk (J.K.); petra.priscakova@fmed.uniba.sk (P.P.); helena.gbelcova@fmed.uniba.sk (H.G.); abolfazl.heydari@savba.sk (A.H.); 2 Institute of Medical Biology, Genetics and Clinical Genetics, Faculty of Medicine, Comenius University, 811 08 Bratislava, Slovakia; 3 Polymer Institute of the Slovak Academy of Sciences, Dúbravská Cesta 9, 845 41 Bratislava, Slovakia; 4 Department of Urology, Faculty of Medicine, Comenius University, Limbová 5, 833 05 Bratislava, Slovakia

**Keywords:** bioadhesive, injectable, hydrogel, stem cell, differentiation

## Abstract

Injectable bioadhesive hydrogels, known for their capacity to carry substances and adaptability in processing, offer great potential across various biomedical applications. They are especially promising in minimally invasive stem cell-based therapies for treating cartilage damage. This approach harnesses readily available mesenchymal stem cells (MSCs) to differentiate into chondrocytes for cartilage regeneration. In this review, we investigate the relationship between bioadhesion and MSC differentiation. We summarize the fundamental principles of bioadhesion and discuss recent trends in bioadhesive hydrogels. Furthermore, we highlight their specific applications in conjunction with stem cells, particularly in the context of cartilage repair. The review also encompasses a discussion on testing methods for bioadhesive hydrogels and direct techniques for differentiating MSCs into hyaline cartilage chondrocytes. These approaches are explored within both clinical and laboratory settings, including the use of genetic tools. While this review offers valuable insights into the interconnected aspects of these topics, it underscores the need for further research to fully grasp the complexities of their relationship.

## 1. Introduction

Hydrogels, which are 3D cross-linked natural or synthetic polymer networks with high water-absorbing capacity and versatile fabrication characteristics, have wide-ranging applications, particularly in the fields of tissue engineering (TE) and regenerative medicine [1].

Injectable hydrogels specially offer potential advantages in minimally invasive local drug delivery, precise and site-specific implantation, as well as targeted delivery to hard-to-reach tissue sites and interface tissues. The phase transition in a polymer solution, from liquid to solid at a critical point, is known as the sol–gel transition state. Injectable hydrogels, including in situ forming and shear-thinning hydrogels, undergo a rapid sol–gel phase transition, which allows the matrix an easy taking of the shape of the cavity, providing a suitable fit and interface in tissues [2,3,4]. In this light, the adhesivity of applied hydrogel is one of the crucial properties for hydrogels in biomedicine.

Bioadhesive hydrogels have emerged as pivotal materials in the realm of cell therapy research, owing to their exceptional attributes. These attributes, including desired biocompatibility, biodegradability, tissue and cellular adhesion capabilities, as well as mechanical properties conducive to the emulation of the extracellular matrix (ECM), play a pivotal role in fostering critical cellular processes such as proliferation, wound healing, and tissue regeneration [5,6,7]. Drawing upon the information presented thus far and the observed experimental outcomes, it can be cautiously inferred that hydrogels exhibit favorable attributes as a potential material for biomedical applications, notably hinting at their potential suitability as a conducive environment for the proliferation of stem cells [8,9]. 

In the current scientific landscape, it is notable that there is a lack of recent reviews specifically focusing on the interplay between the bioadhesive properties of hydrogels and their role in the induction of MSCs differentiation. This absence of literature motivates our study for this significant correlation, which deserves attention. While the extant literature does contain a number of reviews that discuss hydrogels in the context of stem cells [10,11], none of them describes the relationship we explore in this review. We intend to examine the existing knowledge regarding hydrogel bioadhesiveness and its correlation to MSC differentiation, with a particular emphasis on elucidating the underlying mechanisms of bioadhesion and MSC proliferation and differentiation. This work is the first in this field due to its unique focus on revealing the multifaceted relationship between bioadhesiveness in hydrogel materials and its implications for the induction of MSC proliferation.

## 2. Bioadhesion

**Figure 1:**
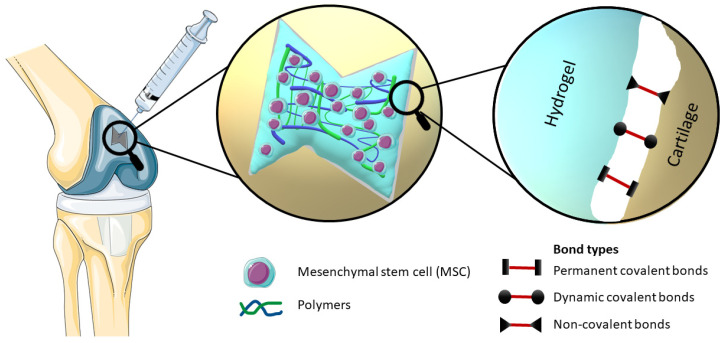
Elucidation of the mechanism of an injectable bioadhesive hydrogel with incorporated stem cells, which effectively occupies the defect in the cartilage structure, provides a visual representation of the different types of bonds involved in bioadhesion, namely Permanent covalent bonds, Dynamic covalent bonds and Non-covalent bonds.

By definition, bioadhesion is the phenomenon in which natural and synthetic materials adhere to biological surfaces. This may or may not be associated with the use of adhesives to bond the material to the biological surface. Bioadhesion also refers to the incorporation of a biomaterial into the body, manifested by the formation of a biofilm on the biomaterial. Xiong at al. divided bioadhesion into three aspects: mucosal adhesion, cell adhesion and bioadhesives [7]. Mucoadhesion is a specific type of bioadhesion in which a layer of mucus gel forms on the surface of the biological surface during the adhesion process [12]. Cell adhesion is a complex phenomenon where aside from morphology, the chemical composition of the biomaterial surface interacts with surface molecules on cells [13]. Bioadhesives derived either from synthetic or biological source are highly biocompatible and biodegradable polymers, which are used to join two surfaces where at least one of them is a living tissue [14]. There are also other approaches to classify bioadhesion. Chopra et al. proposed three types: Type 1: adhesion between two biological phases; Type 2: adhesion of a biological phase to an artificial substrate; Type 3: adhesion of an artificial material to a biological substrate [15]. Overall, hydrogel adhesion involves a complex interplay of chemistry, topology, and mechanics, as various types of bonds are introduced (Table 1 or Figure 1). Hydrogels can manifest robust adhesion through the involvement of both covalent and noncovalent bonds. Covalent bonds contribute their inherent strength individually, while noncovalent bonds, through the synergistic interplay of polymer chains, collectively impart substantial adhesive properties [7,16,17]. The nature of bonds present within hydrogels significantly influences the process of cross-linking, which subsequently impacts their adhesive properties. Notably, heightened cross-linking levels tend to diminish the adhesive capacity of hydrogels. This reduction is attributed to the constrained mobility resulting from increased cross-linking, thereby impeding functional groups along polymer chains from accessing the hydrogel surface and establishing interactions with the substrate for adhesion [18].

Generally, bioadhesives can be classified into three basic categories: I. Wound closure, II. Sealing leakage, III. Immobilization [19]. An ideal bioadhesive polymer is characterized by the following criteria [15]:

1. the polymer and its degradation products must be non-toxic, biodegradable and non-absorbable;
2. it should have the ability to establish robust bonds with mucus or other biological surfaces;
3. rapid and strong adhesion to surfaces should be achievable;
4. it should offer ease of formulation with drugs without impacting drug release patterns.

An overview of recent research developments in the realm of bioadhesive hydrogels (Table 2) collectively offers insights into a variety of applications and material enhancements, underscoring the ongoing progress in the field of bioadhesive hydrogel technology. Then, a more narrowly focused overview of recent research in bioadhesive and bioadhesive injectable hydrogels in cartilage applications is presented in Table 3. 

## 3. Testing of Bioadhesion

Adhesion represents a multifaceted phenomenon governed by intricate interactions involving chemical, topological, and mechanical factors. The comprehensive evaluation of adhesion typically encompasses four distinct mechanical assessments. Notably, within this set of tests, the probe-pull and lap-shear methodologies serve to quantitatively assess adhesion strength by specifically gauging the maximum force per unit area. Meanwhile, the peel and bilayer-stretch tests are employed to assess adhesion toughness, quantifying the energy necessary for separation per unit area. These four tests serve to investigate and differentiate various facets of adhesion properties [16,53].

The majority of adhesion and bioadhesion tests are typically mechanical tests conducted usually ex vivo. Peel tests are a type of mechanical tests used to assess the strength of adhesive bonds, particularly for flexible adherents [54]. There are more variants based on the peeling angle, and all are standardized protocols; e.g., Wei at al. followed standard protocol for peeling adhesion test ASTM F2256-05 [55,56], sometimes minorly modified by research teams; e.g., Jeon et al. utilized a 90° peel test with a porcine skin substrate [57].

One of the most commonly employed assessments for evaluating the adhesive properties of bioadhesive hydrogels involves the utilization of the lap-shear test, also referred to as bulk adhesion testing. The test assesses shear strength, with cohesive failure occurring within the adhesive, while the adhesive failure depends on the adherend’s interface properties [58]. It is a standardized method (ASTM F2255:2005 [59]) that research teams modify [60]; usually, the test undergoes ex vivo utilizing porcine skin [20,61], and the test could be also performed in vitro [41].

The form of a bilayer stretch test methodology can be applied to assess extensional adhesion, wherein the adhesion energy is quantified when the hydrogels are either in their unextended or extended states [62]. Moreover, novel perspectives on adhesion measurement are emerging; e.g., Dehene et al. recently introduced a straightforward and replicable supplement method in viable tissues [63]. Ultimately, scientific teams frequently quantify adhesion in a straightforward manner by using weights and increasing tensile loading till adhesion failure [64,65].

In addition to mechanical tests, biocompatibility tests are an important part of hydrogel bioadhesiveness tests. One such test is the ISO-10993-11 [66] medical device rules and standards. Thanusha at al. evaluated biocompatibility for the developed hydrogel wound dressing [67].

The evaluation of bioadhesive hydrogels also includes clinical trials. For instance, as reported in the study by Øvrebø et al., the transition of hydrogels from laboratory development to clinical application necessitates adherence to an extensive array of protocols and regulatory standards, as well as the establishment of post-market surveillance measures [68].

## 4. Application of Bioadhesive Injectable Hydrogels in Cartilage Regeneration

Bioadhesive injectable hydrogels have garnered substantial interest in recent years due to their remarkable properties. The diverse applications of these hydrogels, ranging from wound healing and tissue repair to cell adhesion and wearable sensors, are discussed, underscoring their promising role in biomedicine and offering valuable insights for future research [7]. As an illustration of the increasing prominence of bioadhesive injectable hydrogels in medicine, various studies stand out. These studies encompass adhesive hydrogels for delivering mesenchymal stem cell-derived exosomes to treat spinal cord injuries [69], an innovative approach using hypoxia-stimulated exosomes within a peptide-modified adhesive hydrogel for spinal cord injury treatment [70], the GelMA–dopamine–EV hydrogel for enhanced MSC-EV function in diabetic wound healing [71], and an adhesive hydrogel integrated with placental mesenchymal stem cell-conditioned medium (CM) to prevent uterine adhesions and improve patient outcomes [72]. Additionally, a PEG-based hydrogel shows potential for muscle regeneration [73], and Col/APG hydrogels incorporating umbilical cord stem cell factor (SCF) offer effective therapeutic treatment for diabetic wounds [12] as well as for diabetic ulcer treatment [74]. The bioadhesive injectable hydrogel with a phenolic nanozyme (SAN) and a CpGODN adjuvant holds promise for localized immunomodulation and catalytic immunotherapy in the tumor microenvironment [31]. Inspired by mussel adhesive proteins, a dopamine-modified poly(α,β-aspartic acid) derivative (PDAEA) forms an injectable bioadhesive hydrogel with strong adhesion and drug delivery potential [75]. An innovative dynamic cross-linked photothermal hydrogel adhesive exhibits photothermal effects and on-demand removability, suitable for wound closure and healing, including MRSA-infected wounds [76]. A novel injectable acacia gum (AG) hydrogel with rapid gelation, self-healing, and effective bioadhesion holds promise for future biomedical applications as a wound-healing agent carrier [77]. Lastly, a composite hydrogel designed for bladder injuries shows potential for tissue engineering and bladder tissue regeneration [41], and a Tetra-PEG hydrogel bioadhesive (SS) offers sutureless repair of GI defects with controlled inflammation and tissue regeneration [78].

Articular cartilage has limited regenerative capacity. MSC-based approaches have emerged as a promising alternative in the treatment of cartilage defects and osteoarthritis. MSCs are a promising source of therapeutically relevant cells for hyaline cartilage regeneration due to their capacity to differentiate into the chondrogenic lineage. However, experimental evidence suggests that after a while, intra-articularly injected MSCs tend to differentiate into transient cartilage that is transformed into bone by the endochondral ossification rather than hyaline articular cartilage. This process leads to decreased effectiveness of the treatment. Similarly, the stratified ultrastructure and spatial organization of native hyaline cartilage disappear [79]. At the same time, the most MSCs injected intra-articularly fail to attach to the damaged cartilage layer, and it is possible that they quickly spread into systemic circulation due to the rapid turnover of synovial capillaries and lymphatic vessels [80]. Consequently, for the optimization of clinical strategies in the domain of cell-based cartilage engineering, it becomes imperative to establish a conducive 3D microenvironment. This microenvironment should comprise a tailored amalgamation of biomaterials and bioactive factors, aimed at further augmenting the differentiation of MSCs into chondrocytes.

Mesenchymal stem cells (MSC) are a promising source of therapeutically relevant cells for hyaline cartilage regeneration due to their capacity to differentiate into the chondrogenic lineage. The aim of the targeted differentiation is to obtain an artificial cartilage tissue with biomechanical properties similar to that of native hyaline cartilage (hyperelastic and dissipative properties, smoothness, toughness, wear resistance, resistance to compressive, tensile, and shear forces). In addition to the MSC differentiation into chondrocytes, the enhancement of the synthesis of the proteins of the hyaline cartilage extracellular matrix including fibronectin, collagens, glycosaminoglycans, proteoglycans, cytokines, and growth factors involved in the functioning of cartilage [81,82] is necessary.

**Figure 2:**
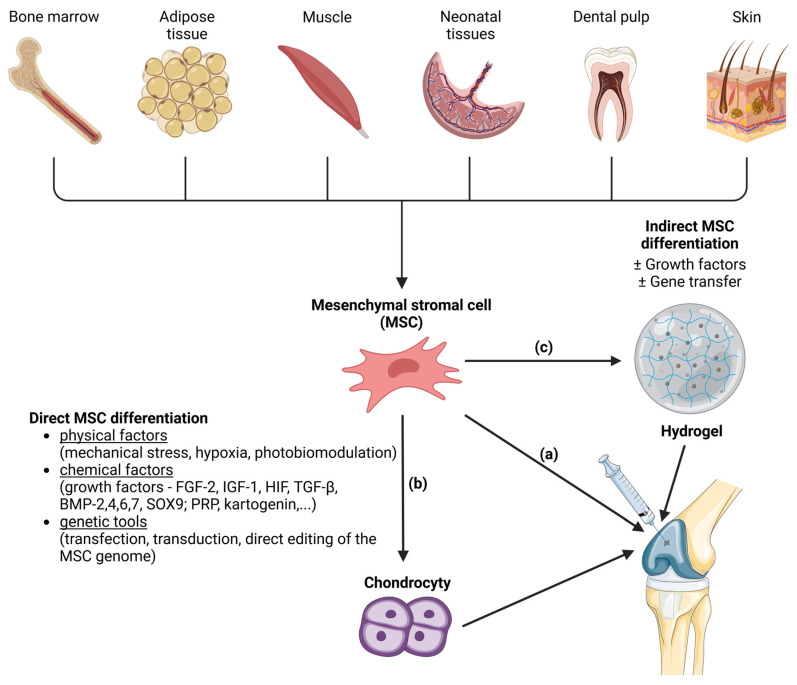
Methods of mesenchymal stem cell (MSC) differentiation into the hyaline cartilage´s chondrocytes. MSCs are a source of the therapeutically relevant cells for cartilage regeneration. MSCs can be harvested from various tissues (bone marrow, adipose tissue, muscle, neonatal tissues, dental pulp and skin). MSCs can be injected directly into joint (**a**). Another approach is induction of MSC differentiation into chondrocytes before microinjection into the joint. Induction of differentiation can be direct (**b**) or indirect (**c**). Direct MSC differentiation can be induced by various physical, chemical and genetical factors. In the case of indirect MSC differentiation, the MSCs are applied to a suitable scaffold (like hydrogels) where the differentiation is induced with or without the presence of growth factors or gene transfer. After that, hydrogel with attached cells is microinjected into the joint. Created with BioRender.com.

MSCs can be applied to a suitable scaffold without prior induction of differentiation. Then, the so-called indirect method of differentiation is carried out, and its success depends on the properties of the scaffold. Another method is the in vitro targeted direct differentiation of MSCs into the chondrocytes that are subsequently applied to the scaffold (Figure 2). Clinical trials with MSC therapies for the regeneration of hyaline cartilage are summarized by Carneiro et al. [83]. Most experimental methods of hyaline cartilage regeneration, which have already been introduced in clinical practice, use direct modification techniques.

## 5. The Clinically Used Direct Methods of the MSC Differentiation into the Hyaline Cartilage’s Chondrocytes

Various physical and chemical factors can be used to induce differentiation of the MSCs into the hyaline cartilage’s chondrocytes. The main physical factors affecting the proliferation of chondrogenic cells are periodic mechanical stress, like cyclic strain or fluid shear stress [84,85,86], hypoxia [87], and electromagnetic radiation, so-called photobiomodulation [88,89,90,91]. Subsequent transformation of the physical signal into a biochemical signal is mediated by integrins and focal adhesion [92]. The potential of feasible hydrostatic pressure to effectively promote the proliferation and chondrogenic differentiation of mesenchymal stem cells was demonstrated in vitro [93], and the effect of photobiomodulation was successfully preclinically [94] as well as clinically [95] tested. Moreover, the combination of the physical stimuli with a scaffold that mimics the native cartilage microenvironment has been found to enhance chondrogenesis for cartilage repair [96].

From the chemical factors, growth factors are an integral part of forming the real conditions of the microenvironment and play a key role in the processes of cell development, including chondrogenic differentiation. Modification of MSCs using recombinant growth factors is one of the simplest, safest, and experimentally and clinically proven approaches to induce differentiation and changes in cell proliferation. The most studied factors with a direct influence on the chondrogenic differentiation of mesenchymal stem cells are fibroblast growth factors FGF-2 [97], insulin-like growth factor IGF-1 [98], hypoxia factors HIFs, cytokines of the transforming growth factor superfamily TGF-β [99,100], and associated bone morphogenetic proteins BMP-2,4,6,7 [101], as well as the SOX9 transcription factor [91,102,103]. A preclinical study on a rabbit cartilage injury model demonstrated the ability of recombinant SOX9 protein to induce reparative tissue formation with features of hyaline cartilage when administered at the site of microfracture [104]. The clinical study focused on the safety and tolerability as well as the dose-limiting toxicity and the maximally tolerated dose of intra-articular BMP-7 finished in 2010, and provided support for the continued development for the treatment of osteoarthritis [105]. However, the results from the phase II studies were not published, and no further studies have been proposed. Currently, there are no BMP-2 products in clinical trials for the treatment of osteoarthritis [106].

The application of platelet-rich plasma (PRP) is another therapeutic approach. Activated platelets (by thrombin, calcium, or collagen) release growth factors that induce chondrogenic differentiation of MSCs that leads to cartilage regeneration in rabbits [107]. Phase I and phase II clinical trials to assess the effectiveness of PRP in the treatment of knee osteoarthritis are ongoing (ClinicalTrials.gov ID: NCT05579665, ClinicalTrials.gov ID NCT02118519).

Small molecule drugs, such as kartogenin (KGN), curcumin, and resveratrol, show promising advantages over frequently used growth factors. They are too small to induce the immune response and are more affordable [108]. KGN is one of the most common small molecules used as a chondrogenic factor, and it is also able to improve the production of chondrogenesis-related proteins of MSCs, including collagen type II and aggrecan [109,110].

## 6. Genetic Tools Used for the Direct MSC Differentiation into the Hyaline Cartilage’s Chondrocytes In Vivo and In Vitro

By genetic modification of cells, which includes transfection, transduction, and direct editing of the genome, it is possible to control chondrogenic differentiation as well as the production of ECM proteins. All three methods have been used to repair cartilage in animals, but have not yet been used in clinical practice. For the application of genetic engineering tools, it is necessary to know in detail the molecular mechanisms of chondrogenic differentiation of MSCs. The topic of signalling pathways and cytokines in chondrogenic differentiation of MSCs is summarized by Yang X et al. [111]. Transgenic growth factors are used for induction of chondrogenic differentiation, transgenic transcription factors are used for the regulation of differentiation, and oligonucleotide delivery can direct MSC differentiation through post-transcriptional gene regulation. Unlike transgenic proteins and RNA, DNA must be carried into the nucleus. From a number of existing methods, those suitable for the modification of a larger population of cells should be selected. Similar to chemical factors, genetic tools allow the use of direct and indirect methods of MSC differentiation; therefore, it is necessary to decide whether the MSC differentiation should take place prior to scaffold seeding, or whether scaffold containing plasmid DNA (pDNA) should be used.

In the process of transduction adenoviruses, retroviruses, herpes simplex virus, adeno-associated viruses, and lentiviruses have been used as vectors to induce chondrogenic differentiation of MSCs [112]. Through transduction, genetic material (vector and transgene) can be delivered directly into the joint (in vivo procedure) or into the explants of cells taken from the joint (ex vivo procedure), whose safety is checked before redelivering into the joint [113,114]. The scientific literature contains many successful examples of MSC transduction to enhance chondrogenesis by increasing the expression of genes responsible for hyaline cartilage proliferation performed either in vitro or in vivo [115]. Although there have been few successful clinical studies in terms of the using transduction for cartilage regeneration [116,117,118], there have also been several clinical cases of the development of tumorigenesis, namely leukemia, after transduction [119]. Therefore, other methods of gene therapy for cartilage regeneration are being investigated for clinical practice.

Primary cell lines, including MSCs and chondrocytes, can be transfected by electroporation [120], or nanocarrier materials, including lipofectamine [119,121,122,123]. The delivery of nucleic acid directly into the nucleus is called nucleofection [124]. Nucleofection is possible by microinjection or by nanocarriers if a nuclear localization sequence is introduced together [125]. Microinjection and nanocarriers are not suitable for large populations of cells [126]. For the elimination of intracellular inflammation and transgene silencing, it has been proven suitable to deliver the minicircle DNA (mcDNA) prepared from pDNA by removing bacterial sequences [127].

The advantage of plasmids is simple preparation and chemical stability, the disadvantage is the necessity of their transcription. In contrast, mRNA and oligonucleotide (small interfering siRNA and micro miRNA) transfection does not require nuclear transport and transcription [128,129]. Similar to DNA and mRNA, siRNA and miRNA can be delivered via nanocarriers [128]. Preclinical and clinical studies on gene therapy for the repair of articular cartilage are reviewed by Bellavia et al. [130].

Another approach of the gene modification of MSCs to enhance chondrogenesis is the knockout of genes in combination with miRNAs that affect the expression of ECM genes; namely, the overexpression of miR-140, miR-21 and miR-675 can stimulate chondrogenesis in MSC cells [131]. New possibilities for the effective treatment of osteoarthritis and other degenerative joint diseases are provided by the relatively new genome editing method CRISPR/Cas [132,133,134]. Through knockout, the CRISPR/Cas technology ensures the activation of a specific gene expression. This approach was successfully applied to the production of ECM proteins in hADSC cells [135] and activation-mediated synthesis of type II collagen and aggrecan in hMSC cells [136].

The use of viral transduction of MSCs for clinical use requires experience in working with viruses and cell cultures and increases the cost of the technique. Transfection, although allowing limited clinical application, leads to low efficiency and high cytotoxicity of MSCs. The application of CRISPR/Cas-mediated gene therapy in clinical practice is limited, in addition to ethical problems, by the possibility of introducing additional, unwanted mutations into the genome. Aspects of genetic modification of MSCs for hyaline cartilage repair are reviewed by Le H et al. [109].

Treatment approaches that incorporate MSCs (even genetically modified), growth factors, and growth-promoting substrates into biocompatible scaffolds can help improve cartilage regeneration [137]. MSCs (or other cells) and growth factors can be applied to the defect site by inserting a three-dimensional (3D) scaffold that promotes growth factor release, cell adhesion, proliferation, and differentiation [138,139]. There are even several promising studies combining the effect of physical (hypoxia) and chemical (growing factors) factors with genetic tools (RNA interference) on MSCs in a 3D scaffold [140,141]. The importance of appropriate stiffness and adhesion of the gene-activated scaffolds that can enhance transfection and chondrogenesis of MSCs has also been confirmed [142].

In the context of employing bioadhesive hydrogels for cartilage injury treatment, innovative approaches include a hybrid photo-cross-linkable (HPC) hydrogel, showcasing fast gelation, robust strength, and tissue adhesion for arthroscopic cartilage repair, with potential as an autologous chondrocyte implantation scaffold [143]. Another study explores an injectable, highly adhesive hydrogel with exosomes for early-stage osteoarthritis cartilage defects, offering potential for minimal cartilage defect treatment and stem cell-based repair [52]. Further research focuses on triple network (TN) hydrogels mimicking cartilage properties, relevant for stem cell-based cartilage repair [144]. Additionally, a mussel-inspired adhesive hydrogel (PDA/Gel-PAA) exhibits high mechanical strength, adhesion, and stem cell-friendly cartilage repair [45]. Lastly, a double cross-linked (DC) hydrogel shows promise for enhancing stem cell-based cartilage repair with improved mechanical properties and anti-degradation characteristics [49]. Building upon the information provided in the preceding referenced studies and publications, it can be reasonably postulated that bioadhesion plays a significant role in the application of hydrogels within the realm of biomedical and stem cell-related research.

## 7. Correlation between Bioadhesion and Stem Cell Differentiation

Cells respond to mechanical cues provided by materials serving as scaffolds. For instance, stiffer hydrogels promote faster growth of neural stem cells [145], while hydrogels with weaker cross-linking facilitate cell migration, favoring regeneration. Adhesion in such environments enhances the exchange of necessary molecules, ultimately improving regeneration [146].

Also, pluripotent stem cell heterogeneity is influenced by mechanical factors related to cell–cell and cell–matrix adhesion. Researchers have experimentally manipulated the spatial polarization of cell–cell adhesion, identifying E-cadherin as one of as key regulators in the differentiation process [147]. The above findings point to a mechanistic role of adhesive materials and of hydrogels in controlling stem cell proliferation and differentiation.

Materials with functional groups can impact cell behavior and stem cell differentiation, often associated with cell spreading. A study employed a unique surface patterning technique to explore the effects of various functional groups (-CH3, -OH, -COOH, -NH2) on mesenchymal stem cells during chondrogenic induction. It was observed that the type of functional groups had an indirect influence on cell differentiation, primarily through protein adsorption, non-specific cell adhesion, and subsequent cell spreading [148].

Surface chemistry of biomaterials has been widely recognized for its role in modulating human mesenchymal stem cell (hMSC) differentiation along specific lineages [149,150]. In an innovative approach, researchers introduced functional groups (acrylic acid and phosphates) onto silk surfaces to direct hMSC differentiation into chondrocytes and osteocytes. Notably, this method does not rely on the addition of growth factors or external signals; differentiation is initiated by the distinct surface functional groups [151].

In addition, controlling cell–polymer interactions is a crucial aspect of scaffold development for tissue engineering. One study investigated the influence of adhesion ligand spacer arm length, such as the RGD peptide coupled to alginate hydrogels, on stem cell behavior. It was found that the spacer arm’s length played a key role in regulating stem cell proliferation and differentiation within polymer scaffolds, holding significance for tissue engineering applications [152]. These findings collectively underscore the complex mechanisms by which adhesion/bioadhesion influence stem cell proliferation and differentiation in tissue engineering contexts.

## 8. Future Perspective

By 2023, 15 randomized controlled clinical trials and 11 nonrandomized RCTs using culture-expanded MSCs in the treatment of knee OA were finished [153]. A total of 179 are ongoing [154], and they suggest net positive effects of MSCs on mitigating pain and symptoms and on cartilage protection and/or repair. In 2023, there are 14 finished or ongoing clinical trials using hydrogels as potential therapies for the management of OA [154]. Adhesive hydrogels improve therapeutic outcomes through offering stable integration between tissue and implants. A total of 133 clinical studies are currently testing effect of platelet-rich plasma and 24 explore the usage of gene therapy in the treatment of OA. Application of hydrogel, MSCs or chemical factors can induce cartilage regeneration, but only to some extent. Many deal with a short half-life and easy clearance from the intended site, leading to low bioavailability. Hydrogels have been shown to influence cell functionality in both chondrocytes and MSCs promoting chondrogenesis and the formation of hyaline cartilage-like ECM. The addition of specific growth factors or gene transfer to hydrogel-encapsulated MSCs may influence cell functionality in cartilage regeneration.

Thus, the combination of adhesive hydrogels, MSC differentiation inductors (growth factors, gene transfer) and MSCs may improve chondrogenic differentiation, maintain chondrogenic phenotype and decrease hypertrophic differentiation in seeded cells while providing optimal biocompatibility, stability, and biodegradability. The combination with bioactive substances and MSCs may help to overcome the hurdle of rapid degradation in natural hydrogels, which often takes place faster than hydrogels can be replaced by de novo ECM [155]. To our knowledge, the combined effectiveness of MSC-laden hydrogels with growth factors/gene transfer has been tested only on animal models (summarized in [155]), but so far with promising outcomes in cartilage regenerations. Next essential steps, as clinical trials, are much needed.

## 9. Conclusions

In conclusion, our comprehensive review underscores the potential correlation between bioadhesive injectable hydrogels and MSC differentiation in the context of cartilage repair. The reviewed literature strongly suggests that these hydrogels hold promise as a promising platform to enhance the regenerative capacity of MSCs for cartilage tissue regeneration. However, further research and clinical studies are imperative to validate and optimize this innovative approach for effective clinical applications.

## Figures and Tables

**Table 1:** Overview of representative chemistry bonds that link hydrogel to biological surfaces known as bioadhesiveness [16].

Bond Types	Representative Bonds
Permanent covalent bonds	Carbon–carbon, Siloxane, Amide, Carbon–nitrogen
Dynamic covalent bonds	Disulfide, Imine, Boronate ester complexations
Non-covalent bonds	Ionic interactions, Hydrogen bonds, Hydrophobic interaction, Dipole–dipole interaction, π–π interaction

**Table 2:** An overview in recent research developments in bioadhesive hydrogels.

Materials	Adhesiveness Origins	Application	Ref.
Acrylic acid and -N,N′-methylenebisacrylamide (MBA) as a cross-linking agent	Phenol groups	Acceleration of oral wound healing	[20]
Vonoprazan fumarate (VF) and acidic fibroblast growth factor (AFGF)	Nano-silica	Hydrogel for Stomach Perforation Repair	[21]
Thiolated γ-polyglutamic acid (PGA-Cys)	Disulfide	For local delivery of keratinocyte growth factor (KGF)	[22]
Poly(acrylic acid) grafted with N-hydroxysuccinimide ester (PAAc-NHS ester) and gelatin	-	Spherical hydrogel network inhalation for enhanced lung defence (SHIELD) against SARS-CoV-2	[23]
Tyramine-modified hyaluronic acid (HA-Tyr)	Tyrosine-containing extracellular matrix proteins	Cartilage repair	[24]
Ion-activated hydrogel (Natural corneal extracellular matrix and peptide-modified alginate)	-	Corneal regeneration	[25]
Polyvinyl alcohol (PVA), dextran (Dex) and borax	Phenol groups	Effectively activated wound healing	[26]
Poloxamer and thiolated γ-PGA polymer	Glycoproteins groups	Diabetic wound healing	[27]
Nitrobenzene-modified hyaluronic acid (HA-NB) + methacrylated polyvinyl alcohol (PVA-MA), with lithium phenyl-2,4,6-trimethylbenzoylphosphinate (LAP) as the photoinitiator	Aldehyde groups	Repair of arterial bleeding	[28]
The protocatechuic aldehyde hybridized collagen-based all-natural hydrogel (FGMA/FG/PA)	Protocatechuic aldehyde (PA)	Promotes Angiogenesis and Diabetic Wound Healing	[29]
Cathechol-conjungated chitosan (CHI-C)	Catechol groups	Hemostasis and bone regeneration	[30]
Catechol-grafted carbon-quantum-dot DA-CQD@Pd hydrogel	Catechol groups	Cancer immunotherapy	[31]
Elastic protein-based hydrogel—grafted glycidyl methacrylate on the gelatin backbone (GELGYM)	-	Ocular tissue engineering	[5]
GTT-3 hydrogel: Tannic acid modified gelatin (Gel-TA) with transglutaminase (TG)	Hydrogen bonding, imine linking, and acyl-transfer reaction	Tissue wound hemostasis	[32]
Methacrylate-hybridizedpoly(3,4-ethylenedioxythiophene)nanoparticle(dPEDOTNP)-incorporated hydrogel	Catechol groups	Brain–machine interference (BMI)	[33]
Chondroitin sulfate (CS), a cartilage-derived sulfated glycosaminoglycan (GAG) as the backbone + catechol moities 3,4-dihydroxyphenylalanine (DOPA)	Catechol groups	Multi-functional bioadhesive	[34]
Platelet-rich plasma (PRP)-laden GelMA hydrogel	-	Hydrogel Contact Lens for the Treatment of Ocular Surface Chemical Injuries	[35]
Silk fibroin (SF) + Poly(ethylene glycol) (PEG) hydrogel	Catechol groups	Wound closure	[36]
Methacryloylated gelatin (GelMA), Pluronic F127 diacrylate (F127DA) & Aldehyded Pluronic F127 (AF127) co-assembled bi-functional micelles and collagen type I (COL I) hydrogel	Aldehyde groups	Corneal patch for in situ sutureless corneal repair	[37]
Cephalexin NT and chitosane hydrogel	Positively charged groups	Antibacterial drug delivery	[38]
Hybrid double-network polydopamine–hyaluronic acid hydrogel	-	Disposable wound dressing	[39]
Gelatin and glycerine-based hydrogel	-	Site-specific drug release	[40]
Methacrylate gelatine + methacrylated silk fibroin + Pluronic F127 diacrylate	Amine-MA and thiol-MA groups plus intermolecular non-covalent bonds	Bladder injury repair	[41]

**Table 3:** An overview in recent research developments in bioadhesive and bioadhesive-injectable hydrogels in cartilage repair.

Materials	Adhesiveness Origins	Ref.
poly(ethyleneglycol)(PEG)–basedhydrogel	Sulfate and acrylate groups.	[42]
Polydopamine–chondroitin sulfate–polyacrylamide (PDA–CS–PAM)	Catechol group	[43]
Catechol-functionalized Chondoritin sulfate (CS-CA) hydrogel	Catechol group	[44]
Polydopamine/gelatin-poly(acrylic acid) (PDA/Gel-PAA) composite hydrogel	Catechol groups	[45]
Oxidized hyaluronic acid (OHA) and N-(2-hydroxypropyl)-3-trimethylammonium chitosan chloride (HTCC) methacrylate (HTCCMA) hydrogel	Aldehyde groups	[46]
Hyaluronic acid-transglutaminase (HA-TG) hydrogel	Covalent bonds	[47]
Self-cross-linked oxidized alginate/gelatin hydrogel	Aldehyde groups	[48]
Doublle cross-linked Hyalourinoc acid hydrogel (Deial Alder clic reaction ad phenyl-boronate ester bond)	Catechol groups	[49]
Tyramine-modified hyaluronic acid (HA-Tyr) hydrogel	Adhesion to cartilage due to hyaluronic acid	[24]
Sulfhydryl chondroitin sulfate and polydopamine (CS-PDA)	Catechol groups	[50]
Gelatine–Methacryloyl(GelMA)–glycol chitosan hydrogel	Protein and methacrylate groups	[51]
Eznymytically cross-linked alginate-–dopamine, chondroitin sulfate, and regenerated silk fibroin (AD/CS/RSF)	Aldehyde and dopamine groups	[52]

## Data Availability

Not applicable.

## References

[1] Wang W., Narain R., Zeng H. (2020). Hydrogels. Polymer Science and Nanotechnology.

[2] Guvendiren M., Lu H.D., Burdick J.A. (2011). Shear-thinning hydrogels for biomedical applications. Soft Matter.

[3] Rizzo F., Kehr N.S. (2020). Recent Advances in Injectable Hydrogels for Controlled and Local Drug Delivery. Adv. Health Mater..

[4] Sun Y., Nan D., Jin H., Qu X. (2019). Recent advances of injectable hydrogels for drug delivery and tissue engineering applications. Polym. Test..

[5] Sharifi S., Islam M.M., Sharifi H., Islam R., Koza D., Reyes-Ortega F., Alba-Molina D., Nilsson P.H., Dohlman C.H., Mollnes T.E. (2021). Tuning gelatin-based hydrogel towards bioadhesive ocular tissue engineering applications. Bioact. Mater..

[6] Chang M., Liu X., Wang X., Peng F., Ren J. (2021). Mussel-inspired adhesive hydrogels based on biomass-derived xylan and tannic acid cross-linked with acrylic acid with antioxidant and antibacterial properties. J. Mater. Sci..

[7] Xiong Y., Zhang X., Ma X., Wang W., Yan F., Zhao X., Chu X., Xu W., Sun C. (2021). A review of the properties and applications of bioadhesive hydrogels. Polym. Chem..

[8] Stan D., Tanase C., Avram M., Apetrei R., Mincu N.B., Mateescu A.L., Stan D. (2021). Wound healing applications of creams and ‘smart’ hydrogels. Exp. Dermatol..

[9] Dey K., Agnelli S., Serzanti M., Ginestra P., Scarì G., Dell’Era P., Sartore L. (2019). Preparation and properties of high performance gelatin-based hydrogels with chitosan or hydroxyethyl cellulose for tissue engineering applications. Int. J. Polym. Mater. Polym. Biomater..

[10] Yazdani M., Shahdadfar A., Jackson C.J., Utheim T.P. (2019). Hyaluronan-Based Hydrogel Scaffolds for Limbal Stem Cell Transplantation: A Review. Cells.

[11] Li Z., Yue M., Liu Y., Zhang P., Qing J., Liu H., Zhou Y. (2022). Advances of Engineered Hydrogel Organoids within the Stem Cell Field: A Systematic Review. Gels.

[12] Kheyraddini Mousavi A., Leseman Z.C., Palacio M.L.B. (2012). Bioadhesion. Encyclopedia of Nanotechnology.

[13] Palacio M.L.B., Bhushan B. (2012). Bioadhesion: A review of concepts and applications. Philos. Trans. R. Soc. A Math. Phys. Eng. Sci..

[14] Uma K. (2023). Bioadhesives for clinical applications—A mini review. Mater Adv..

[15] Chopra H., Kumar S., Singh I. (2020). Bioadhesive Hydrogels and Their Applications. Bioadhesives in Drug Delivery.

[16] Yang J., Bai R., Chen B., Suo Z. (2020). Hydrogel Adhesion: A Supramolecular Synergy of Chemistry, Topology, and Mechanics. Adv. Funct. Mater..

[17] Yao H., Wu M., Lin L., Wu Z., Bae M., Park S., Wang S., Zhang W., Gao J., Wang D. (2022). Design strategies for adhesive hydrogels with natural antibacterial agents as wound dressings: Status and trends. Mater. Today Bio..

[18] Li Z., Yu C., Kumar H., He X., Lu Q., Bai H., Kim K., Hu J. (2022). The Effect of Crosslinking Degree of Hydrogels on Hydrogel Adhesion. Gels.

[19] Duan W., Bian X., Bu Y. (2021). Applications of Bioadhesives: A Mini Review. Front. Bioeng. Biotechnol..

[20] Sun J., Chen T., Zhao B., Fan W., Shen Y., Wei H., Zhang M., Zheng W., Peng J., Wang J. (2023). Acceleration of Oral Wound Healing under Diabetes Mellitus Conditions Using Bioadhesive Hydrogel. ACS Appl. Mater. Interfaces.

[21] Liu S., Luan Z., Wang T., Xu K., Luo Q., Ye S., Wang W., Dan R., Shu Z., Huang Y. (2023). Endoscopy Deliverable and Mushroom-Cap-Inspired Hyperboloid-Shaped Drug-Laden Bioadhesive Hydrogel for Stomach Perforation Repair. ACS Nano.

[22] Xu H.L., Tong M.Q., Wang L.F., Chen R., Li X.Z., Sohawon Y., Zhao Y.Z. (2019). Thiolated γ-polyglutamic acid as a bioadhesive hydrogel-forming material: Evaluation of gelation, bioadhesive properties and sustained release of KGF in the repair of injured corneas. Biomater. Sci..

[23] Mei X., Li J., Wang Z., Zhu D., Huang K., Hu S., Cheng K. (2023). An inhaled bioadhesive hydrogel to shield non-human primates from SARS-CoV-2 infection. Nat. Mater..

[24] Behrendt P., Ladner Y., Stoddart M.J., Lippross S., Alini M., Eglin D., Armiento A.R. (2020). Articular Joint-Simulating Mechanical Load Activates Endogenous TGF-β in a Highly Cellularized Bioadhesive Hydrogel for Cartilage Repair. Am. J. Sports Med..

[25] Zhao L., Shi Z., Sun X., Yu Y., Wang X., Wang H., Li T., Zhang H., Zhang X., Wang F. (2022). Natural Dual-Crosslinking Bioadhesive Hydrogel for Corneal Regeneration in Large-Size Defects. Adv. Healthc. Mater..

[26] Xie X., Lei Y., Li Y., Zhang M., Sun J., Zhu M.-Q., Wang J. (2023). Dual-crosslinked bioadhesive hydrogel as NIR/pH stimulus-responsiveness platform for effectively accelerating wound healing. J. Colloid Interface Sci..

[27] Yao Q., Shi Y., Xia X., Tang Y., Jiang X., Zheng Y.-W., Zhang H., Chen R., Kou L. (2021). Bioadhesive hydrogel comprising bilirubin/β-cyclodextrin inclusion complexes promote diabetic wound healing. Pharm. Biol..

[28] Luo S., Yang L., Zou Q., Yuan D., Xu S., Zhao Y., Wu X., Wang Z., Ye C. (2023). Rapid suture-free repair of arterial bleeding: A novel approach with ultra-thin bioadhesive hydrogel membrane. Chem. Eng. J..

[29] Fu Y., Shi Y., Wang L., Zhao Y., Wang R., Li K., Zhang S., Zha X., Wang W., Zhao X. (2023). All-Natural Immunomodulatory Bioadhesive Hydrogel Promotes Angiogenesis and Diabetic Wound Healing by Regulating Macrophage Heterogeneity. Adv. Sci..

[30] Huang W., Cheng S., Wang X., Zhang Y., Chen L., Zhang L. (2021). Noncompressible Hemostasis and Bone Regeneration Induced by an Absorbable Bioadhesive Self-Healing Hydrogel. Adv. Funct. Mater..

[31] He H., Fei Z., Guo T., Hou Y., Li D., Wang K., Ren F., Fan K., Zhou D., Xie C. (2022). Bioadhesive injectable hydrogel with phenolic carbon quantum dot supported Pd single atom nanozymes as a localized immunomodulation niche for cancer catalytic immunotherapy. Biomaterials.

[32] Zhou J., Wu Y., Zhang X., Lai J., Li Y., Xing J., Teng L., Chen J. (2021). Enzyme Catalyzed Hydrogel as Versatile Bioadhesive for Tissue Wound Hemostasis, Bonding, and Continuous Repair. Biomacromolecules.

[33] Wang X., Sun X., Gan D., Soubrier M., Chiang H.-Y., Yan L., Li Y., Li J., Yu S., Xia Y. (2022). Bioadhesive and conductive hydrogel-integrated brain-machine interfaces for conformal and immune-evasive contact with brain tissue. Matter.

[34] Zhu W., Iqbal J., Wang D.-A. (2018). A DOPA-functionalized chondroitin sulfate-based adhesive hydrogel as a promising multi-functional bioadhesive. J. Mater. Chem. B.

[35] Soykan M.N., Altug B., Bas H., Ghorbanpoor H., Avci H., Eroglu S., Sengel S.B., Sariboyaci A.E., Bagis S.G., Uysal O. (2023). Developing a Novel Platelet-Rich Plasma-Laden Bioadhesive Hydrogel Contact Lens for the Treatment of Ocular Surface Chemical Injuries. Macromol. Biosci..

[36] Liu H., Yuan M., Sonamuthu J., Yan S., Huang W., Cai Y., Yao J. (2020). A dopamine-functionalized aqueous-based silk protein hydrogel bioadhesive for biomedical wound closure. New J. Chem..

[37] Li M., Wei R., Liu C., Fang H., Yang W., Wang Y., Zhou X. (2023). A ‘T.E.S.T.’ hydrogel bioadhesive assisted by corneal cross-linking for in situ sutureless corneal repair. Bioact. Mater..

[38] Salatin S., Jelvehgari M. (2020). Desirability function approach for development of a thermosensitive and bioadhesive nanotransfersome–hydrogel hybrid system for enhanced skin bioavailability and antibacterial activity of cephalexin. Drug Dev. Ind. Pharm..

[39] Gao Y.-M., Li Z.-Y., Zhang X.-J., Zhang J., Li Q.-F., Zhou S.-B. (2023). One-Pot Synthesis of Bioadhesive Double-Network Hydrogel Patch as Disposable Wound Dressing. ACS Appl. Mater. Interfaces.

[40] Cassano R., Curcio F., Mandracchia D., Trapani A., Trombino S. (2020). Gelatin and Glycerine-Based Bioadhesive Vaginal Hydrogel. Curr. Drug Deliv..

[41] Fu Z., Xiao S., Wang P., Zhao J., Ling Z., An Z., Shao J., Fu W. (2023). Injectable, stretchable, toughened, bioadhesive composite hydrogel for bladder injury repair. RSC Adv..

[42] Sharma B., Fermanian S., Gibson M., Unterman S., Herzka D.A., Cascio B., Coburn J., Hui A.Y., Marcus N., Gold G.E. (2013). Human Cartilage Repair with a Photoreactive Adhesive-Hydrogel Composite. Sci. Transl. Med..

[43] Han L., Wang M., Li P., Gan D., Yan L., Xu J., Wang K., Fang L., Chan C.W., Zhang H. (2018). Mussel-Inspired Tissue-Adhesive Hydrogel Based on the Polydopamine–Chondroitin Sulfate Complex for Growth-Factor-Free Cartilage Regeneration. ACS Appl. Mater. Interfaces.

[44] Shin J., Kang E.H., Choi S., Jeon E.J., Cho J.H., Kang D., Lee H., Yun I.S., Cho S.-W. (2021). Tissue-Adhesive Chondroitin Sulfate Hydrogel for Cartilage Reconstruction. ACS Biomater. Sci. Eng..

[45] Yan L., Zhou T., Ni R., Jia Z., Jiang Y., Guo T., Wang K., Chen X., Han L., Lu X. (2022). Adhesive Gelatin-Catechol Complex Reinforced Poly(Acrylic Acid) Hydrogel with Enhanced Toughness and Cell Affinity for Cartilage Regeneration. ACS Appl. Bio Mater..

[46] Qiu H., Deng J., Wei R., Wu X., Chen S., Yang Y., Gong C., Cui L., Si Z., Zhu Y. (2023). A lubricant and adhesive hydrogel cross-linked from hyaluronic acid and chitosan for articular cartilage regeneration. Int. J. Biol. Macromol..

[47] Levinson C., Cavalli E., von Rechenberg B., Zenobi-Wong M., Darwiche S.E. (2021). Combination of a Collagen Scaffold and an Adhesive Hyaluronan-Based Hydrogel for Cartilage Regeneration: A Proof of Concept in an Ovine Model. Cartilage.

[48] Balakrishnan B., Joshi N., Jayakrishnan A., Banerjee R. (2014). Self-crosslinked oxidized alginate/gelatin hydrogel as injectable, adhesive biomimetic scaffolds for cartilage regeneration. Acta Biomater..

[49] Yu C., Gao H., Li Q., Cao X. (2020). Injectable dual cross-linked adhesive hyaluronic acid multifunctional hydrogel scaffolds for potential applications in cartilage repair. Polym. Chem..

[50] Li D., Li J., Chen T., Qin X., Pan L., Lin X., Liang W., Wang Q. (2023). Injectable Bioadhesive Hydrogels Scavenging ROS and Restoring Mucosal Barrier for Enhanced Ulcerative Colitis Therapy. ACS Appl. Mater. Interfaces.

[51] Paul S., Schrobback K., Tran P.A., Meinert C., Davern J.W., Weekes A., Klein T.J. (2023). Photo-Cross-Linkable, Injectable, and Highly Adhesive GelMA-Glycol Chitosan Hydrogels for Cartilage Repair. Adv. Healthc. Mater..

[52] Zhang F.-X., Liu P., Ding W., Meng Q.-B., Su D.-H., Zhang Q.-C., Lian R.-X., Yu B.-Q., Zhao M.-D., Dong J. (2021). Injectable Mussel-Inspired highly adhesive hydrogel with exosomes for endogenous cell recruitment and cartilage defect regeneration. Biomaterials.

[53] Wang M., Wang C. (2019). Bulk Properties of Biomaterials and Testing Techniques. Encyclopedia of Biomedical Engineering.

[54] Ebnesajjad S., Landrock A.H. (2015). Testing of Adhesive Bonds. Adhesives Technology Handbook.

[55] Wei K., Senturk B., Matter M.T., Wu X., Herrmann I.K., Rottmar M., Toncelli C. (2019). Mussel-Inspired Injectable Hydrogel Adhesive Formed under Mild Conditions Features Near-Native Tissue Properties. ACS Appl. Mater. Interfaces.

[56] (2015). Standard Test Method for Strength Properties of Tissue Adhesives in T-Peel by Tension Loading.

[57] Jeon E.Y., Joo K.I., Cha H.J. (2020). Body temperature-activated protein-based injectable adhesive hydrogel incorporated with decellularized adipose extracellular matrix for tissue-specific regenerative stem cell therapy. Acta Biomater..

[58] Mathias J.-D., Grédiac M., Michaud P. (2016). Bio-based adhesives. Biopolymers and Biotech Admixtures for Eco-Efficient Construction Materials.

[59] (2005). Standard Test Method for Strength Properties of Tissue Adhesives in Lap-Shear by Tension Loading.

[60] Jung H.Y., Le Thi P., HwangBo K.-H., Bae J.W., Park K.D. (2021). Tunable and high tissue adhesive properties of injectable chitosan based hydrogels through polymer architecture modulation. Carbohydr. Polym..

[61] Sigen A., Xu Q., Johnson M., Creagh-Flynn J., Venet M., Zhou D., Wang W. (2021). An injectable multi-responsive hydrogel as self-healable and on-demand dissolution tissue adhesive. Appl. Mater. Today.

[62] Yang J., Steck J., Bai R., Suo Z. (2020). Topological adhesion II. Stretchable adhesion. Extreme. Mech. Lett..

[63] Dehne T., Zehbe R., Krüger J.P., Petrova A., Valbuena R., Sittinger M., Schubert H., Ringe J. (2012). A method to screen and evaluate tissue adhesives for joint repair applications. BMC Musculoskelet. Disord..

[64] Zhu F., Wang C., Yang S., Wang Q., Liang F., Liu C., Qiu D., Qu X., Hu Z., Yang Z. (2017). Injectable tissue adhesive composite hydrogel with fibroblasts for treating skin defects. J. Mater. Chem. B.

[65] Chen S., Tomov M.L., Ning L., Gil C.J., Hwang B., Bauser-Heaton H., Chen H., Serpooshan V. (2023). Extrusion-Based 3D Bioprinting of Adhesive Tissue Engineering Scaffolds Using Hybrid Functionalized Hydrogel Bioinks. Adv. Biol..

[66] (2017). Biological Evaluation of Medical Devices. Part 11: Tests for Systemic Toxicity.

[67] Thanusha A.V., Koul V. (2022). Biocompatibility evaluation for the developed hydrogel wound dressing—ISO-10993-11 standards—In vitro and in vivo study. Biomed. Phys. Eng. Express.

[68] Øvrebø Ø., Perale G., Wojciechowski J.P., Echalier C., Jeffers J.R., Stevens M.M., Rossi F. (2022). Design and clinical application of injectable hydrogels for musculoskeletal therapy. Bioeng. Transl. Med..

[69] Li L., Zhang Y., Mu J., Chen J., Zhang C., Cao H., Gao J. (2020). Transplantation of Human Mesenchymal Stem-Cell-Derived Exosomes Immobilized in an Adhesive Hydrogel for Effective Treatment of Spinal Cord Injury. Nano Lett..

[70] Mu J., Li L., Wu J., Huang T., Zhang Y., Cao J., Ma T., Chen J., Zhang C., Zhang X. (2022). Hypoxia-stimulated mesenchymal stem cell-derived exosomes loaded by adhesive hydrogel for effective angiogenic treatment of spinal cord injury. Biomater. Sci..

[71] Wang Y., Song P., Wu L., Su Z., Gui X., Gao C., Zhou C. (2023). In situ photo-crosslinked adhesive hydrogel loaded with mesenchymal stem cell-derived extracellular vesicles promotes diabetic wound healing. J. Mater. Chem. B.

[72] Zhu Y., Wang T., Bao M.-J., Qu X.-H., Li Z.-M. (2023). Effect of stem cell conditional medium-loading adhesive hydrogel on TGF-β1-induced endometrial stromal cell fibrosis. Front. Bioeng. Biotechnol..

[73] Han W.M., Mohiuddin M., Anderson S.E., García A.J., Jang Y.C. (2019). Co-delivery of Wnt7a and muscle stem cells using synthetic bioadhesive hydrogel enhances murine muscle regeneration and cell migration during engraftment. Acta Biomater..

[74] Zhang L., Zhou Y., Su D., Wu S., Zhou J., Chen J. (2021). Injectable, self-healing and pH responsive stem cell factor loaded collagen hydrogel as a dynamic bioadhesive dressing for diabetic wound repair. J. Mater. Chem. B.

[75] Gong C., Lu C., Li B., Shan M., Wu G. (2017). Injectable dopamine-modified poly(α,β-aspartic acid) nanocomposite hydrogel as bioadhesive drug delivery system. J. Biomed. Mater. Res. A.

[76] Kang X., Guan P., Xiao C., Liu C., Guan Y., Lin Y., Tian Y., Ren K., Huang Y., Fu R. (2023). Injectable Intrinsic Photothermal Hydrogel Bioadhesive with On-Demand Removability for Wound Closure and MRSA-Infected Wound Healing. Adv. Health Mater..

[77] Ahmadian Z., Jelodar M.Z., Rashidipour M., Dadkhah M., Adhami V., Sefareshi S., Ebrahimi H.A., Ghasemian M., Adeli M. (2023). A self-healable and bioadhesive acacia gum polysaccharide-based injectable hydrogel for wound healing acceleration. DARU J. Pharm. Sci..

[78] Li S., Xian Y., He G., Chen L., Chen Z., Hong Y., Wu D. (2023). In situ Injectable Tetra-PEG Hydrogel Bioadhesive for Sutureless Repair of Gastrointestinal Perforation. Chin. J. Chem..

[79] Somoza R.A., Welter J.F., Correa D., Caplan A.I. (2014). Chondrogenic Differentiation of Mesenchymal Stem Cells: Challenges and Unfulfilled Expectations. Tissue Eng. Part B Rev..

[80] Huang J., Liu Q., Xia J., Chen X., Xiong J., Yang L., Liang Y. (2022). Modification of mesenchymal stem cells for cartilage-targeted therapy. J. Transl. Med..

[81] Fuentes-Mera L., Camacho A., Moncada-Saucedo N.K., Peña-Martínez V. (2017). Current Applications of Mesenchymal Stem Cells for Cartilage Tissue Engineering. Mesenchymal Stem Cells—Isolation, Characterization and Applications.

[82] Danišovič Ľ., Boháč M., Zamborský R., Oravcová L., Provazníková Z., Csöbönyeiová M., Varga I. (2016). Comparative analysis of mesenchymal stromal cells from different tissue sources in respect to articular cartilage tissue engineering. Gen. Physiol. Biophys..

[83] Carneiro D.D.C., Araújo L.T.D., Santos G.C., Damasceno P.K.F., Vieira J.L., Santos R.R.D., Soares M.B.P. (2023). Clinical Trials with Mesenchymal Stem Cell Therapies for Osteoarthritis: Challenges in the Regeneration of Articular Cartilage. Int. J. Mol. Sci..

[84] Huang X., Das R., Patel A., Duc Nguyen T. (2018). Physical Stimulations for Bone and Cartilage Regeneration. Regen. Eng. Transl. Med..

[85] Shahmoradi S.R., Kabir Salmani M., Soleimanpour H.R., Tavakoli A.H., Hosaini K., Haghighipour N., Bonakdar S. (2019). Induction of Chondrogenic Differentiation in Human Mesenchymal Stem Cells Cultured on Human Demineralized Bone Matrix Scaffold under Hydrostatic Pressure. Tissue Eng. Regen. Med..

[86] Dusfour G., Maumus M., Cañadas P., Ambard D., Jorgensen C., Noël D., Le Floc’H S. (2020). Mesenchymal stem cells-derived cartilage micropellets: A relevant in vitro model for biomechanical and mechanobiological studies of cartilage growth. Mater. Sci. Eng. C.

[87] Gómez-Leduc T., Desancé M., Hervieu M., Legendre F., Ollitrault D., de Vienne C., Herlicoviez M., Galéra P., Demoor M. (2017). Hypoxia Is a Critical Parameter for Chondrogenic Differentiation of Human Umbilical Cord Blood Mesenchymal Stem Cells in Type I/III Collagen Sponges. Int. J. Mol. Sci..

[88] Parate D., Franco-Obregón A., Fröhlich J., Beyer C., Abbas A.A., Kamarul T., Hui J.H.P., Yang Z. (2017). Enhancement of mesenchymal stem cell chondrogenesis with short-term low intensity pulsed electromagnetic fields. Sci. Rep..

[89] Marks R. (2021). Articular Cartilage Degradation and Photobiomodulation Therapy. CPQ Orthop..

[90] Schneider C., Dungel P., Priglinger E., Danzer M., Schädl B., Nürnberger S. (2021). The impact of photobiomodulation on the chondrogenic potential of adipose-derived stromal/stem cells. J. Photochem. Photobiol. B Biol..

[91] Bozhokin M.S., Vcherashnii D.B., Yastrebov S.G., Beilinson L.L., Zherebtsova Ju V., Khotin M.G. (2022). Low-intensity photobiomodulation at 632.8 nm increases tgfβ3, col2a1, and sox9 gene expression in rat bone marrow mesenchymal stem cells in vitro. Lasers Med. Sci..

[92] Potter C.M.F., Lao K.H., Zeng L., Xu Q. (2014). Role of Biomechanical Forces in Stem Cell Vascular Lineage Differentiation. Arter. Thromb. Vasc. Biol..

[93] Cheng B., Tu T., Shi X., Liu Y., Zhao Y., Zhao Y., Li Y., Chen H., Chen Y., Zhang M. (2019). A novel construct with biomechanical flexibility for articular cartilage regeneration. Stem Cell Res. Ther..

[94] Fekrazad R., Eslaminejad M.B., Shayan A.M., Kalhori K.A., Abbas F.M., Taghiyar L., Pedram M.S., Ghuchani M.S. (2016). Effects of Photobiomodulation and Mesenchymal Stem Cells on Articular Cartilage Defects in a Rabbit Model. Photomed. Laser Surg..

[95] Ferraresi C., Freire F., Hamblin M. (2018). Photobiomodulation in Cartilage: In vitro, in vivo, and Clinical Trials. Low-Level Light Therapy: Photobiomodulation.

[96] Sawatjui N., Limpaiboon T., Schrobback K., Klein T. (2018). Biomimetic scaffolds and dynamic compression enhance the properties of chondrocyte- and MSC -based tissue-engineered cartilage. J. Tissue Eng. Regen. Med..

[97] Khan W.S., Tew S.R., Adesida A.B., Hardingham T.E. (2008). Human infrapatellar fat pad-derived stem cells express the pericyte marker 3G5 and show enhanced chondrogenesis after expansion in fibroblast growth factor-2. Thromb. Haemost..

[98] Armakolas N., Dimakakos A., Armakolas A., Antonopoulos A., Koutsilieris M. (2016). Possible role of the Ec peptide of IGF-1Ec in cartilage repair. Mol. Med. Rep..

[99] Jia Z., Wang S., Liang Y., Liu Q. (2019). Combination of kartogenin and transforming growth factor-β3 supports synovial fluid-derived mesenchymal stem cell-based cartilage regeneration. Am. J. Transl. Res..

[100] Monaco G., El Haj A.J., Alini M., Stoddart M.J. (2020). Sodium Hyaluronate Supplemented Culture Media as a New hMSC Chondrogenic Differentiation Media-Model for in vitro/ex vivo Screening of Potential Cartilage Repair Therapies. Front. Bioeng. Biotechnol..

[101] Fu H., Wang H., Li D. (2017). BMP-7 accelerates the differentiation of rabbit mesenchymal stem cells into cartilage through the Wnt/β-catenin pathway. Exp. Ther. Med..

[102] Kozhemyakina E., Lassar A.B., Zelzer E. (2015). A pathway to bone: Signaling molecules and transcription factors involved in chondrocyte development and maturation. Development.

[103] Gibson J.D., O’sullivan M.B., Alaee F., Paglia D.N., Yoshida R., Guzzo R.M., Drissi H. (2017). Regeneration of Articular Cartilage by Human ESC-Derived Mesenchymal Progenitors Treated Sequentially with BMP-2 and Wnt5a. Stem Cells Transl. Med..

[104] Zhang X., Wu S., Naccarato T., Prakash-Damani M., Chou Y., Chu C.-Q., Zhu Y. (2017). Regeneration of hyaline-like cartilage in situ with SOX9 stimulation of bone marrow-derived mesenchymal stem cells. PLoS ONE.

[105] Hunter D.J., Pike M.C., Jonas B.L., Kissin E., Krop J., McAlindon T. (2010). Phase 1 safety and tolerability study of BMP-7 in symptomatic knee osteoarthritis. BMC Musculoskelet. Disord..

[106] Whitty C., Pernstich C., Marris C., McCaskie A., Jones M., Henson F. (2022). Sustained delivery of the bone morphogenetic proteins BMP-2 and BMP-7 for cartilage repair and regeneration in osteoarthritis. Osteoarthr. Cartil. Open.

[107] Chen Y.-C., Hsu Y.-M., Tan K.P., Fang H.-W., Chang C.-H. (2018). Intraarticular injection for rabbit knee osteoarthritis: Effectiveness among hyaluronic acid, platelet-rich plasma, and mesenchymal stem cells. J. Taiwan Inst. Chem. Eng..

[108] Feng X., Li J., Zhang X., Liu T., Ding J., Chen X. (2019). Electrospun polymer micro/nanofibers as pharmaceutical repositories for healthcare. J. Control. Release.

[109] Le H., Xu W., Zhuang X., Chang F., Wang Y., Ding J. (2020). Mesenchymal stem cells for cartilage regeneration. J. Tissue Eng..

[110] Spakova T., Plsikova J., Harvanova D., Lacko M., Stolfa S., Rosocha J. (2018). Influence of Kartogenin on Chondrogenic Differentiation of Human Bone Marrow-Derived MSCs in 2D Culture and in Co-Cultivation with OA Osteochondral Explant. Molecules.

[111] Yang X., Tian S., Fan L., Niu R., Yan M., Chen S., Zheng M., Zhang S. (2022). Integrated regulation of chondrogenic differentiation in mesenchymal stem cells and differentiation of cancer cells. Cancer Cell Int..

[112] Chen F.H., Rousche K.T., Tuan R.S. (2006). Technology Insight: Adult stem cells in cartilage regeneration and tissue engineering. Nat. Clin. Pract. Rheumatol..

[113] Steinert A.F., Nöth U., Tuan R.S. (2008). Concepts in gene therapy for cartilage repair. Injury.

[114] Li K.-C., Hu Y.-C. (2015). Cartilage Tissue Engineering: Recent Advances and Perspectives from Gene Regulation/Therapy. Adv. Healthc. Mater..

[115] Rodriguez-Merchan E.C., Valentino L.A. (2019). The Role of Gene Therapy in Cartilage Repair. Arch. Bone Jt. Surg..

[116] Ha C.-W., Noh M.J., Choi K.B., Lee K.H. (2012). Initial phase I safety of retrovirally transduced human chondrocytes expressing transforming growth factor-beta-1 in degenerative arthritis patients. Cytotherapy.

[117] Kim M.K., Ha C.W., In Y., Cho S.D., Choi E.S., Ha J.K., Lee M.C. (2018). A Multicenter, Double-Blind, Phase III Clinical Trial to Evaluate the Efficacy and Safety of a Cell and Gene Therapy in Knee Osteoarthritis Patients. Hum. Gene Ther. Clin. Dev..

[118] Lee B., Parvizi J., Bramlet D., Romness D.W., Guermazi A., Noh M., Sodhi N., Khlopas A., Mont M.A. (2020). Results of a Phase II Study to Determine the Efficacy and Safety of Genetically Engineered Allogeneic Human Chondrocytes Expressing TGF-β1. J. Knee Surg..

[119] Hamann A., Nguyen A., Pannier A.K. (2019). Nucleic acid delivery to mesenchymal stem cells: A review of nonviral methods and applications. J. Biol. Eng..

[120] Bucher C., Gazdhar A., Benneker L.M., Geiser T., Gantenbein-Ritter B. (2013). Nonviral Gene Delivery of Growth and Differentiation Factor 5 to Human Mesenchymal Stem Cells Injected into a 3D Bovine Intervertebral Disc Organ Culture System. Stem Cells Int..

[121] Kelly A.M., Plautz S.A., Zempleni J., Pannier A.K. (2016). Glucocorticoid Cell Priming Enhances Transfection Outcomes in Adult Human Mesenchymal Stem Cells. Mol. Ther..

[122] Park J.S., Yi S.W., Kim H.J., Kim S.M., Kim J.-H., Park K.-H. (2017). Construction of PLGA Nanoparticles Coated with Polycistronic *SOX5*, *SOX6*, and *SOX9* Genes for Chondrogenesis of Human Mesenchymal Stem Cells. ACS Appl. Mater. Interfaces.

[123] de Carvalho T.G., Pellenz F.M., Laureano A., Silla L.M.d.R., Giugliani R., Baldo G., Matte U. (2018). A simple protocol for transfecting human mesenchymal stem cells. Biotechnol. Lett..

[124] Nakashima S., Matsuyama Y., Nitta A., Sakai Y., Ishiguro N. (2005). Highly Efficient Transfection of Human Marrow Stromal Cells by Nucleofection. Transplant. Proc..

[125] Hoare M., Greiser U., Schu S., Mashayekhi K., Aydogan E., Murphy M., Barry F., Ritter T., O’Brien T. (2010). Enhanced lipoplex-mediated gene expression in mesenchymal stem cells using reiterated nuclear localization sequence peptides. J. Gene Med..

[126] Han S.-W., Nakamura C., Kotobuki N., Obataya I., Ohgushi H., Nagamune T., Miyake J. (2008). High-efficiency DNA injection into a single human mesenchymal stem cell using a nanoneedle and atomic force microscopy. Nanomedicine.

[127] Mun J.-Y., Shin K.K., Kwon O., Lim Y.T., Oh D.-B. (2016). Minicircle microporation-based non-viral gene delivery improved the targeting of mesenchymal stem cells to an injury site. Biomaterials.

[128] Benoit D.S.W., Boutin M.E. (2012). Controlling Mesenchymal Stem Cell Gene Expression Using Polymer-Mediated Delivery of siRNA. Biomacromolecules.

[129] Levy O., Zhao W., Mortensen L.J., LeBlanc S., Tsang K., Fu M., Karp J.M. (2013). mRNA-engineered mesenchymal stem cells for targeted delivery of interleukin-10 to sites of inflammation. Blood.

[130] Bellavia D., Veronesi F., Carina V., Costa V., Raimondi L., De Luca A., Giavaresi G. (2018). Gene therapy for chondral and osteochondral regeneration: Is the future now?. Cell. Mol. Life Sci..

[131] Gurusinghe S., Strappe P. (2014). Gene Modification of Mesenchymal Stem Cells and Articular Chondrocytes to Enhance Chondrogenesis. BioMed Res. Int..

[132] Adkar S.S., Brunger J.M., Willard V.P., Wu C.-L., Gersbach C.A., Guilak F. (2017). Genome Engineering for Personalized Arthritis Therapeutics. Trends Mol. Med..

[133] Nishimasu H., Shi X., Ishiguro S., Gao L., Hirano S., Okazaki S., Noda T., Abudayyeh O.O., Gootenberg J.S., Mori H. (2018). Engineered CRISPR-Cas9 nuclease with expanded targeting space. Science.

[134] Tanikella A.S., Hardy M.J., Frahs S.M., Cormier A.G., Gibbons K.D., Fitzpatrick C.K., Oxford J.T. (2020). Emerging Gene-Editing Modalities for Osteoarthritis. Int. J. Mol. Sci..

[135] Farhang N., Davis B., Weston J., Ginley-Hidinger M., Gertz J., Bowles R.D. (2020). Synergistic CRISPRa-Regulated Chondrogenic Extracellular Matrix Deposition Without Exogenous Growth Factors. Tissue Eng. Part A.

[136] Huynh N.P., Gloss C.C., Lorentz J., Tang R., Brunger J.M., McAlinden A., Guilak F. (2020). Long non-coding RNA GRASLND enhances chondrogenesis via suppression of the interferon type II signaling pathway. Elife.

[137] Huang H., Hu X., Zhang X., Duan X., Zhang J., Fu X., Dai L., Yuan L., Zhou C., Ao Y. (2018). Codelivery of Synovium-Derived Mesenchymal Stem Cells and TGF-β by a Hybrid Scaffold for Cartilage Regeneration. ACS Biomater. Sci. Eng..

[138] Tsuzuki N., Seo J.P., Yamada K., Haneda S., Furuoka H., Tabata Y., Sasaki N. (2013). The effect of a gelatin β-tricalcium phosphate sponge loaded with mesenchymal stem cells (MSC), bone morphogenic protein-2, and platelet-rich plasma (PRP) on equine articular cartilage defect. Can. Vet. J..

[139] Huynh N.P.T., Brunger J.M., Gloss C.C., Moutos F.T., Gersbach C.A., Guilak F. (2018). Genetic Engineering of Mesenchymal Stem Cells for Differential Matrix Deposition on 3D Woven Scaffolds. Tissue Eng. Part A.

[140] Legendre F., Ollitrault D., Gomez-Leduc T., Bouyoucef M., Hervieu M., Gruchy N., Galéra P. (2017). Enhanced chondrogenesis of bone marrow-derived stem cells by using a combinatory cell therapy strategy with BMP-2/TGF-β1, hypoxia, and COL1A1/HtrA1 siRNAs. Sci. Rep..

[141] Branly T., Bertoni L., Contentin R., Rakic R., Gomez-Leduc T., Desancé M., Galéra P. (2017). Characterization and use of Equine Bone Marrow Mesenchymal Stem Cells in Equine Cartilage Engineering. Study of their Hyaline Cartilage Forming Potential when Cultured under Hypoxia within a Biomaterial in the Presence of BMP-2 and TGF-ß1. Stem Cell Rev. Rep..

[142] Ledo A.M., Vining K.H., Alonso M.J., Garcia-Fuentes M., Mooney D.J. (2020). Extracellular matrix mechanics regulate transfection and SOX9-directed differentiation of mesenchymal stem cells. Acta Biomater..

[143] Hua Y., Xia H., Jia L., Zhao J., Zhao D., Yan X., Zhang Y., Tang S., Zhou G., Zhu L. (2021). Ultrafast, tough, and adhesive hydrogel based on hybrid photocrosslinking for articular cartilage repair in water-filled arthroscopy. Sci. Adv..

[144] Demott C.J., Jones M.R., Chesney C.D., Grunlan M.A. (2023). Adhesive Hydrogel Building Blocks to Reconstruct Complex Cartilage Tissues. ACS Biomater. Sci. Eng..

[145] Liang Y., Li S., Li Y., Li M., Sun X., An J., Xu Q., Chen Z., Wang Y. (2021). Impact of hydrogel stiffness on the induced neural stem cells modulation. Ann. Transl. Med..

[146] Feng Q., Wei K., Lin S., Xu Z., Sun Y., Shi P., Li G., Bian L. (2016). Mechanically resilient, injectable, and bioadhesive supramolecular gelatin hydrogels crosslinked by weak host-guest interactions assist cell infiltration and in situ tissue regeneration. Biomaterials.

[147] Toh Y.-C., Xing J., Yu H. (2015). Modulation of integrin and E-cadherin-mediated adhesions to spatially control heterogeneity in human pluripotent stem cell differentiation. Biomaterials.

[148] Cao B., Peng Y., Liu X., Ding J. (2017). Effects of Functional Groups of Materials on Nonspecific Adhesion and Chondrogenic Induction of Mesenchymal Stem Cells on Free and Micropatterned Surfaces. ACS Appl. Mater. Interfaces.

[149] Amadori S., Torricelli P., Panzavolta S., Parrilli A., Fini M., Bigi A. (2015). Multi-Layered Scaffolds for Osteochondral Tissue Engineering: In Vitro Response of Co-Cultured Human Mesenchymal Stem Cells. Macromol. Biosci..

[150] Dong L., Cheng K., Zhou Y., Yu M., Gong J., Lin Y., Luo Q., Wang Q., Weng W., Wang H. (2017). Surface Atomic Structure Directs the Fate of Human Mesenchymal Stem Cells. ACS Appl. Mater. Interfaces.

[151] Patil S., Singh N. (2018). Spatially controlled functional group grafting of silk films to induce osteogenic and chondrogenic differentiation of human mesenchymal stem cells. Mater. Sci. Eng. C.

[152] Lee J.W., Kim H., Lee K.Y. (2016). Effect of spacer arm length between adhesion ligand and alginate hydrogel on stem cell differentiation. Carbohydr. Polym..

[153] Copp G., Robb K.P., Viswanathan S. (2023). Culture-expanded mesenchymal stromal cell therapy: Does it work in knee osteoarthritis? A pathway to clinical success. Cell. Mol. Immunol..

[154] Duan W.-L., Zhang L.-N., Bohara R., Martin-Saldaña S., Yang F., Zhao Y.-Y., Xie Y., Bu Y.-Z., Pandit A. (2023). Adhesive hydrogels in osteoarthritis: From design to application. Mil. Med. Res..

[155] Wagenbrenner M., Mayer-Wagner S., Rudert M., Holzapfel B.M., Weissenberger M. (2021). Combinations of Hydrogels and Mesenchymal Stromal Cells (MSCs) for Cartilage Tissue Engineering—A Review of the Literature. Gels.

